# Which Health Cares Are Related to the Family Physician? A Critical Interpretive Synthesis of Literature

**Published:** 2017-05

**Authors:** Shahram YAZDANI, Maryam AKBARILAKEH

**Affiliations:** Dept. of Medical Education, School of Medical Education, Shahid Beheshti University of Medical Sciences, Tehran, Iran

**Keywords:** Family practice, Family physician educational program, Iran

## Abstract

**Background:**

This study provided the theoretical basis for program development through a new conceptualization of the concept of family physician related health care.

**Methods:**

Critical interpretive synthesis (CIS) was used to carry out qualitative analysis and synthesis of the literature from 2006 until 2015. At the beginning of CIS, the search strategy was designed to access electronic databases such as CINAHL, Medline, Cochrane library, PsycINFO, Embase, EBMreviews, and Thomson scientific web of science database. The main review question was the clarification of the health care related to family physician in health system, which produced over related 750 articles; 60 articles related to the research objective were studied by purposive sampling. After identifying the main categories and sub-categories, synthesis of the contradictory findings in different studies was conducted. New concepts and relationships between concepts were created using CIS of documentation related to the place of family physician in health system.

**Results:**

To define the original position of family physician in health system, clarify its related health care and determine its boundaries from other health care providers, and its use in the design and development of family physician’s educational program, a frame of concepts related to the main concept and question was created. A more useful means of understanding family physician is offered by the synthetic constructs of this framework.

**Conclusion:**

The theoretical conceptualization of family physician position and duties in the health system can be an appropriate guide for educational program and curricula in our context.

## Introduction

Although comprehensive definition is not provided to health; however, among the definitions provided in general, Health is a state of complete comfort, welfare and wellbeing of physical, mental, social and spiritual (1). Health is not only the absence of disease, disability, and death; discomfort and dissatisfaction, even in the absence of the disease can reduce health. In general, the concept of health encompasses all aspects of human life and is widespread (2).

Several factors affect health such as disease, health risk factors including environmental, behavioral and genetic, social determinants of health including poverty, illiteracy, injustice, etc. Effective access means the possibility of taking services to potential beneficiaries of services, affordability means the price of services, insurance coverage services, financial indigent patients, and the willingness of patients to pay, health literacy means the ability to access, analyze, and understand basic health information to make good decisions for health, treatment seeking behavior means the health care system customers decisions of applying for health care with respect to time, place, and manner, patient compliance behaviors, resources and technology, skills and abilities of service providers, provider incentive mechanisms including external incentive mechanisms such as various methods of payment, professional behavior of service providers including ethics and professionalism, the mechanisms for their intrinsic motivation (3, 4).

Given attention to these factors is a step in the development of quality health care system that is conducive to the proper function of all health care providers. Among service providers, primary health care providers such as Family physicians are the first coordinators of health care system, which is why during the past three decades, interventions related to family physicians, has formed fixed component of health reform in all countries (5). Our country is not an exception and reform of the health system in our country is formed around the concept of family medicine. To set up an educational and operational program for family practice, you need to clarify the part of health care related to family physician in health system to provide, their appropriate roles attribute in the outcomes of the training program.

This study provided the theoretical basis for program development through a new conceptualization of the concept of family physician related health care. The literature on the concept of primary health care providers’ roles attributes including family physician is large, diverse, and complex, especially when deciding to establish an educational program. A review of this area by CIS would be of most benefit.

## Methods

Among many methods of synthesis, interpretive reviews, respect to the induction and interpretation synchronously. CIS approach used to synthesize in qualitative manner as systematic review used for quantitative ones. CIS intended to develop new concepts and theories with a large sample of diverse papers (6). Other methods of review are more limited when the aim, is to generating conceptual frame for a vague concept. However, CIS is suited to the production of interpretive syntheses rather than simple aggregation of data (7). Clarification of the concept of family physicians’ related health cares and re-conceptualizing of it in health system is our main review question. This question specifies the research domain and its original shape gradually becomes complete by the end of the study. This question, due to the research results modified iteratively (8).

At the beginning of CIS, the search strategy designed to access electronic databases such as CINAHL, Medline, Cochrane Library, PsycINFO, Embase, EBM Reviews, and Thomson Scientific Web of Science database, and the Arts and Humanities Citation Index from 2006 till 2015. In this search, we found literature relating to the research question and over 750 articles related to the topic of research; then 60 articles related to the research objective were studied by purposive sampling. Purposive sampling was used initially to select papers and later used theoretical sampling to add, test, and develop the emerging analysis. This project includes each type of research design.

In conventional systematic review we have specific criteria for critically appraising the quality of the articles’ designs, but in CIS prioritizes the papers that appear to be relevant to the research subject, instead of focusing on particular study types or papers with specified methodological standards. Quality assessment is done during analysis and synthesis through value judgments of many discourses and arguments in various articles by researchers. We included the articles that help to develop our own synthetic argument (8).

In both qualitative and quantitative papers, data extraction was done by extracting the headlines of the categories and sub-categories using the terms of the paper itself as well as a summary of the relevant material.

In CIS, interpretive synthesis strategies were done such as meta-ethnography, but with some modifications. Refutational synthesis attempts made a synthesis from contradictions between the findings of articles. The reciprocal translational analysis attempts to translate the concepts into each other and help in order to develop our own synthetic argument from various judgments of discourses and arguments (9).

Lines of argument synthesis involve building a general third order construct grounded in the themes or categories extracted before from separate studies findings that help to clarify the main question (9).

CIS analysis was begun with detailed inspection of the papers, gradually identifying recurring themes and developing a critique. Generated themes helped to explain the health cares related to family physician in health system, by constantly comparing the theoretical structures and categories of our analysis and the relationships between them and synthesis conducted through reconceptualization (8).

Ministry of Health and Medical Education (MOHME) in Iran approved the present study. The codes of ethics related to this research project were respected.

## Results

We found that individuals, enterprises, institutions, resources, and activities exist that their primary or secondary purpose is considering the factors affecting health and health promotion (10). Highlighting the boundary between their health cares’ duties is the basis for our concept clarification.

The terms “primary” and “secondary” only referred to the priority goal of “health promotion” in the list of objectives of the organizations (11). The inter-sectorial organizations such as medical schools, Clinical Research Center, medical diagnostic laboratories, clinics, private offices, hospitals, specialty and etc. the primary objective is to maintain and promote health; and in external organizations such as the Ministry of Education, Ministry of Transportation, Ministry of Agriculture, Islamic Republic of Iran Broadcasting (IRIB), health promotion, is secondary purpose (12). In general, the health sector includes all individuals, organizations, institutions, resources, and activities that their primary goal is protecting and improving health.

Health System refers to a subset of the health sector coordinated in order to achieve the preset objectives. The main objectives of the health system, including health promotion, increase satisfaction levels of society, to minimize injustice in society, and protection of patients and their families against financial pressures caused by the disease (13). Achieving the goals of the health system requires four main functions of service delivery, resource generation, supply and financial allocation, and stewardship. Among these functions, the health system is known for the performance of service delivery (14).

Healthcare System is a subset of the health system to provide directly a range of health services. The task of the healthcare system is that the right patient, at the right time and in the right place, receives the right service by the right provider.

In Iran is an integrated model of health care system that the dominant model is the National System of Health Service (NHS). This means that the services and health facilities supported by the government but along with public health, insurance and social security insurance coverage are broad (15).

Traditionally, the health services provided in the health system are on three levels: Primary Health Care (PHC), Secondary and Tertiary (16).

PHC has included the range of services in order to provide the health and maintain safety of the public; from the promotion of health and screening for disease to assessment, diagnosis, treatment and rehabilitation of patients, as well as social services to people (17). These are the first level of contact of people with the health system and they can access to them through self-referral. Primary care emphasizes on the cooperation of individuals and social groups to promote individual and community health. PHC makes possible to achieve equity, effectiveness, and efficiency of health services. A strong PHC promotes health outcomes (18).

Many health care providers provide services that are part of PHC. However, providing one or more service of PHC will not cause it to be classified as primary care providers. For being classified, as a provider of health services in PHC, must have certain features is needed such as Continuous and Constant contact with individuals and families over time in health and disease. First Contact by self-referral of patients and without limitation; comprehensive covering the full range of services, from education and prevention, diagnosis and treatment and rehabilitation; Members involved in PHC act in coordinated with each other as well as with other service providers in second and third levels; PHC providers act Community Oriented; Family Centered; Culturally Competent. These features are generalized to general practice or family medicine (19).

Over ally, PHC providers include General and Family Practitioners, Public Health Nurses, General Nurses, Social Worriers, Practice Nurses, Midwives, Community Mental Health Nurses, Dieticians, Dentists, Community Welfare Officers, Physiotherapist, Chiropodists, Community Pharmacists, and Psychologists (20).

Usually, a combination of the above as primary care team provides primary care and each of these teams are responsible for specific demographic coverage. Due to the key importance of PHC, in many countries, this practice is defined as an integrated system referred to as PHC delivery system (21).

In addition to primary care, health care system provides other levels of care including specialty and emergency care. Although the boundaries between different levels of care are not fully transparent but through four main indicators can separate these levels of care (22). We discussed them in above (PHC certain features including first contact, continuity, comprehensiveness, coordination), emergency care also includes first contact and to some degree coordination and a little comprehensiveness. In Specialty care, these features are very low (23).

The term “primary care” is a broader sense of general (family) practice and from the other side, general practice is not only limited to primary health services. In fact, primary care is original position which family physician provides services in it. In most cases, general or family practitioners do also part of emergency care. Then the role of general or family practitioners is to see the part of the PHC and emergency care services (24).

**Fig. 1:**
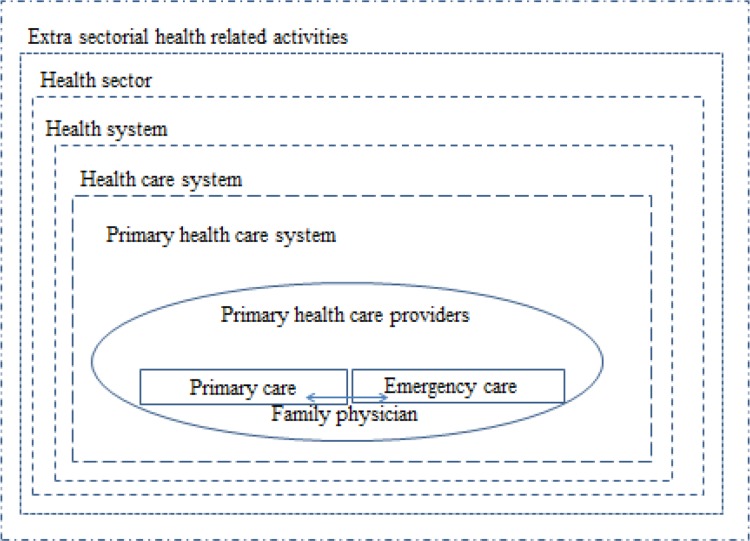
Family physician related health care frame

We should look for educational program due to original position of general or family practitioners. The conceptual model that underpins family physician in the health system shows our interpretive findings and synthesis (Fig. 1).

## Discussion

By CIS approach, we have clarified what are the family physician related health cares in comparison to closely related health care levels and in comparison to other primary health care providers. In our synthesis, the semantic boundaries of family physician educational program’s challenging areas are identified. Our clarification of the family physician’s original position paves the way and solves the challenge of the ambiguity of the meaning of its roles and duties, in health system. The proposed frame covers health system items. This frame is a useful resource and reference for those who want to conduct family physician educational program. This is an absolute, definitive framework to provide a complete instructional design, and these frame items are necessary for developing family physician’s health care outcomes in our health system. Therefore, this initial frame development is a crucial step in the development of family physicians policies, programs, and curricula (25).

This CIS project is the first step in the process of moving toward an operational program development. By such this project, we can move from separated information and a series of facts about something to the conceptual frame understanding the connections and interactions between facts. This understanding should happen in each individual family physician as a part of our health system.

Then the individual is centered in our educational program, and the educational process should happen to the individual. Finally, we must say this frame should continue in order to develop a practical guideline for family physicians’ experiments in our context. Our CIS project is a part of a great work, scheduled by MOHME to take place in our country, to develop the basis for experimental works and to ground and benchmark the theories in practice. We are equipped with this reference framework to develop a practical educational model for family physicians in our context. However, we have lacked this basic research for practical guides to development the instructional program and implementation of it. This study will be useful to start eliminating these deficits and to improve the relationship of our medical education system by our health system.

## Ethical considerations

Ethical issues (Including plagiarism, informed consent, misconduct, data fabrication and/or falsification, double publication and/or submission, redundancy, etc.) have been completely observed by the authors.
